# Ocular involvement in pediatric Behçet’s disease: is it different than in adults? (a short case series and mini review)

**DOI:** 10.1186/s12886-023-03197-5

**Published:** 2023-11-21

**Authors:** Casem Azri, Perrine Dusser, Laura Eid, Emmanuel Barreau, Isabelle Kone-Paut, Charlotte Borocco, Caroline Galeotti, Sami Saad, Marc Labetoulle, Antoine Rousseau

**Affiliations:** 1 Service d’Ophtalmologie, Assistance Publique Hôpitaux de Paris (AP-HP), Université Paris-Saclay. Centre de Référence Pour Les Maladies Rares en Ophtalmologie (OPHTARA), Hôpital Bicêtre, 78, Rue du Général Leclerc, 94275 Le Kremlin Bicêtre, France; 2 Paediatric Rheumatology Department, APHP, Bicêtre Hospital, Le Kremlin-Bicêtre, 94270 France; 3 Centre de Référence Des Maladies Auto-Inflammatoires Et Des Amyloses Inflammatoire (CEREMAIA), Le Kremlin-Bicêtre, France; 4 Service d’Ophtalmologie, Centre Hospitalier National Ophtalmologique des 15-20, Paris, France; 5 Department of Immunology of Viral, Auto-Immune Disease, Hematological and Bacterial Diseases (IMVA-HB), UMR1184, CEA, Fontenay-Aux-Roses, France

**Keywords:** Pediatric Behçet disease, Uveitis, Vasculitis, Biotherapies

## Abstract

**Background:**

Pediatric Behçet’s disease (PBD) is rarer than BD and can be a challenging diagnosis as clinical picture may be incomplete. As in adult patients, sight-threatening ocular manifestations may lead to diagnosis. In this study, we aimed to report a series of cases of PBD with ocular manifestations and provide a review of the literature.

**Methods:**

Retrospective case series of PBD patients with ocular manifestations. Demographic, ophthalmological and systemic data at presentation and during follow-up were collected and analyzed.

**Results:**

Four patients, aged 13.0 ± 2.9 years (9–16) were included. Posterior uveitis with retinal vasculitis, papillitis and macular edema was present in all patients, with associated anterior uveitis in 2 cases. Other features included occlusive vasculitis (2/4) and necrotizing retinitis (2/4). All patients were improved by systemic treatments except one patient with severe bilateral optic neuropathy. Ocular manifestations were the presenting symptoms in 3/4 cases.

**Conclusion:**

Ocular manifestations and systemic associations of PBD are comparable to those encountered in adult patients. The lack of complains in pediatric patients may lead to a longer diagnosis delay, especially in unilateral uveitis. Aggressive and long-term treatment is mandatory to prevent vision loss and recurrences.

## Background

Behcet’s disease (BD) is an immune-mediatedvasculitis involving vessels of all sizes, which presents with multisystemic features and sight-threatening ocular manifestations [1].The course of the disease may be recurrent and refractory, requiring chronic treatments with potential adverse effects [1].

There is a high geographic variation in the incidence rate of BD: most of the countries with a high incidence (20 to 420/100.000) are located along the historical silk road, Turkey having the highest rate in the world [1, 2]. While the peak age of onset is in the young adult population (20–30 years old), onset of BD can occur in children (before 16 years) in 3% to 26% of cases [3–5]. In pediatric Behçet’s disease (PBD), the diagnosis may represent a clinical challenge as it commonly features an incomplete clinical picture [6]. Thus, a definition of PBD was recently proposed by the Consensus classification criteria for pediatric Behçet’s disease study (PEDBD) to take into account these peculiarities [7]. The following typical ocular involvements are included in the diagnostic criteria: anterior uveitis, posterior uveitis and retinal vasculitis [7]. These diagnostic criteria include ophthalmological, neurological and vascular items, but oral aphthosis is not mandatory [7]. As PBD is a rare condition, it is not clear whether ocular manifestations are really different from those encountered in adult patients [8]. We here report a case series of PBD with ocular manifestations and provide a review of the literature about this rare but severe clinical situation.

## Methods

We retrospectively included pediatric patients (Age ≤ 18 y.o. at diagnosis) with Behçet related uveitis, managed at the Department of Ophthalmology in Bicêtre hospital, France, between 2012 and 2020.

BD diagnosis was made using the PEDBD criteria. If the patient did not meet PEDBD criteria, diagnosis could be made using ICBD (International criteria for Behçet’s disease study [9]) criteria or after multidisciplinary agreement. PEDBD and ICBD criteria are available in Table 1. Medical files were used to analyze demographic data (age, ethnicity, medical history), and extra-ocular involvement. Ophthalmological data included initial and final best-corrected visual acuity (BCVA), uveitis classification according to the Standardization of Uveitis Nomenclature (SUN) classification criteria [10] slit lamp and fundus features. Fluorescein angiography and macular optical coherence tomography (OCT) findings were analyzed. Systemic and topical treatments, clinical response, and long-term follow-up were analyzed for each patient. The study was performed according to regulations of the local ethics committee. Institutional Review Board (IRB)/Ethics Committee approval was obtained by the French Society of Ophthalmology (IRB 00008855 Société Française d’Ophtalmologie IRB#1).

**Table 1:** PEDBD criteria and ICBD criteria

**PEDBD criteria** ***Score* ≥ *3 indicates pediatric Behçet’s disease***		
Item	Description	Points
Recurrent oral aphthosis	At least three attacks/year	1
Genital ulceration	Typically with scar	1
Skin involvement	Necrotic folliculitis, acneiform lesions,erythema nodosum	1
Ocular involvement	Anterior uveitis, posterior uveitis, retinal vasculitis	1
Neurological signs	With the exception of isolated headaches	1
Vascular signs	Venous thrombosis, arterial thrombosis,arterial aneurysm	1
**ICBD criteria** ***Score* ≥ *4 indicates Behçet’s diagnosis***		
Item	Description	Points
Oral aphtosis	Not specified	2
Genital aphtosis	Not specified	2
Ocular lesions	Anterior uveitis, posterior uvitis or retinal vasculitis	2
Skin lesions	Pseudofolliculitis, skin aphtosis, erythema nodosum	1
Neurological manifestations	Not specified	1
Vascular manifestations	Arterial thrombosis, large vein thrombosis, phlebitis or superficial phlebitis	1
Positive pathergy test	Not specified	1

*PEDBD* PEDiatric Behçet’s Disease study [7], *ICBD* International Criteria for Behçet’s disease study [9]

## Results

Four patients (3 males, 1 female) aged 13.0 ± 2.9 years (9–16) were included. They were all from North African descent. One patient had a heterozygous mutation of the familial Mediterranean fever gene (MEFV). Oral aphthosis was present in 3 patients, while none had both oral and genital aphtosis. Neurological manifestations were present in 3 cases. Ocular involvement led to the diagnosis of BD in all cases. Three patients met the PEDBD criteria, while 1 patient with typical neurological and ocular involvement was diagnosed as BD after multidisciplinary agreement. Three patients met the ICBD criteria. Presenting ocular symptoms were red eye (2/4) and loss of vision (4/4). Posterior uveitis with retinal vasculitis and papillitis was present in all patients, macular edema was present in 2 cases. Associated anterior uveitis was present in 2 cases. Other features included occlusive vasculitis (2/4), and necrotizing retinitis (2/4). Corticosteroid combined with azathioprine and anti-TNFα were administered in all patients (doses available in Table 2). One patient with severe bilateral optic neuropathy also received cyclophosphamide pulses. He had complete bilateral vision loss caused by optic atrophy during follow-up. Among the 3 remaining, BCVA of the most severe eye improved from 1.5 ± 1.1 (0.2–2.3) at presentation to 0.0 ± 0.1 LogMAR (0–0.1) at the last visit. Mean delay before diagnosis was 11.3 ± 8.5 months (0.3–21). No patient relapsed during follow-up, 63.5 ± 28,6 months (19–104). Detailed results are provided in Table 2.

**Table 2:** Patients characteristics at the onset of uveitis. M = male, F = female, MP = methyliprendisolone

Case #	Age/ gender	Descent	BCVA at presentation (logMar)	Final BCVA (logMAR)	Presenting symptoms	Diagnostic delay (months)	Systemic features	Associated conditions	Anterior / Posterior uveitis	Retinal vasculitis	Ocular Complications	Systemic Treatments	Ophthalmic Treatments	PEDBD criteria fulfilled	ICBD criteria fulfilled	Follow-up (months)
1	13 / M	Northern Africa HLAB51 +	OD: 0 OS: 2.3	OD: 0 OS: 0	Ocular	18	Oral aphtosis pseudofolliulitis	Hétérozygous M694I	- / +	+	Macular edema Serous retinal detachment Necrotizing retinitis Occlusive vasculitis	Intravenous MP pulse 15 mg/kg and oral prednisone Infliximab 5 mg/kg Azathioprine 2 mg/kg/day Colchicine 1 mg/day	Hourly dexamethasone eye drops Tropicamide and phenylephrine eye drops	+	+	69
2	9 / M	Northern Africa HLAB51 +	OD: 0.4 OS: 1.9	OD: 0 OS: 0.1	Ocular	0.3	Oral aphtosis Meningitidis Anorexia Fever	None	+ / +	+	Diffuse capillaritis discrete bilateral papilledema	Intravenous MP pulse 15 mg/kg and oral prednisone Infliximab 5 mg/kg Azathioprine 2 mg/kg/day Colchicine 1 mg/day	Dexamethasone subconjunctival injection Hourly Dexamethasone eyed drops Tropicamide eye drops	+	+	24
3	14 / F	Northern Africa HLAB51 +	OD: 0.2 OS: 0	OD: 0 OS: 0	Neurological: Encephalomyelitis with aseptic meningitis	21	Encephalomyelitis with aseptic meningitis polyarthralgia	- None	-—/ +	+	Macular edema Vasculitis Papillitis	Intravenous MP pulse 15 mg/kg and oral prednisone Infliximab 5 mg/kg Azathioprine 2 mg/kg/day Colchicine 1 mg/day	Dexamethasone eye drops (6/day)	-	-	57
4	16 / M	Northern Africa HLAB51-	OD: 2.7 OS: 0.7	OD: 3 OS: 3	Ocular	6	Oral aphtosis Cerebral venous sinus thrombosis Psychiatric manifestations Anorexia	None	+ / +	+	Ischemic retinopathy with necrotizing retinitis Optic neuritis Occlusive vasculitis	Intravenous MP pulse 15 mg/kg and oral prednisone Infliximab 5 mg/kg Cyclophosphamide 750 mg/day Azathioprine 2 mg/kg/day Colchicine 1 mg/day Enoxaparin 150UI/day	Dexamethasone eye drops (4/Day)	+	+	104

*M* Male, *F* Female, *BCVA* Best corrected visual acuity, *OD* Right eye, *OS* Left eye, *MP* Methylprednisolone, *PEDBD* Pediatric Behçet’s Disease study [7], *ICBD* International Criteria for Behçet’s Disease [9]

## Case 1

A 13-year-old boy, from Moroccan descent, was referred for blurred vision on the left eye. He was heterozygous for the familial Mediterranean fever gene (*M694I*), and HLAB-51-positive. He suffered from recurrent fever and oral aphtosis which was diagnosed as PFAPA (periodic fever, aphthous stomatitis, pharyngitis, adenitis) syndrome from his third to his 7th year and was treated with colchicine. He was then lost to follow-up but reported having had recurrent oral ulcers from the age of 7 without genital involvement for which he had never sought medical attention. At the age of 13 he consulted for ocular pain, redness, and sudden drop in visual acuity of his left eye. He reported 3 previous episodes of red eye and floaters which were treated as conjunctivitis at the ages of 11 and 12. He also reported episodes of pseudo-folliculitis and headaches 1–2 times in the preceding 2 years.

**Fig. 1:**
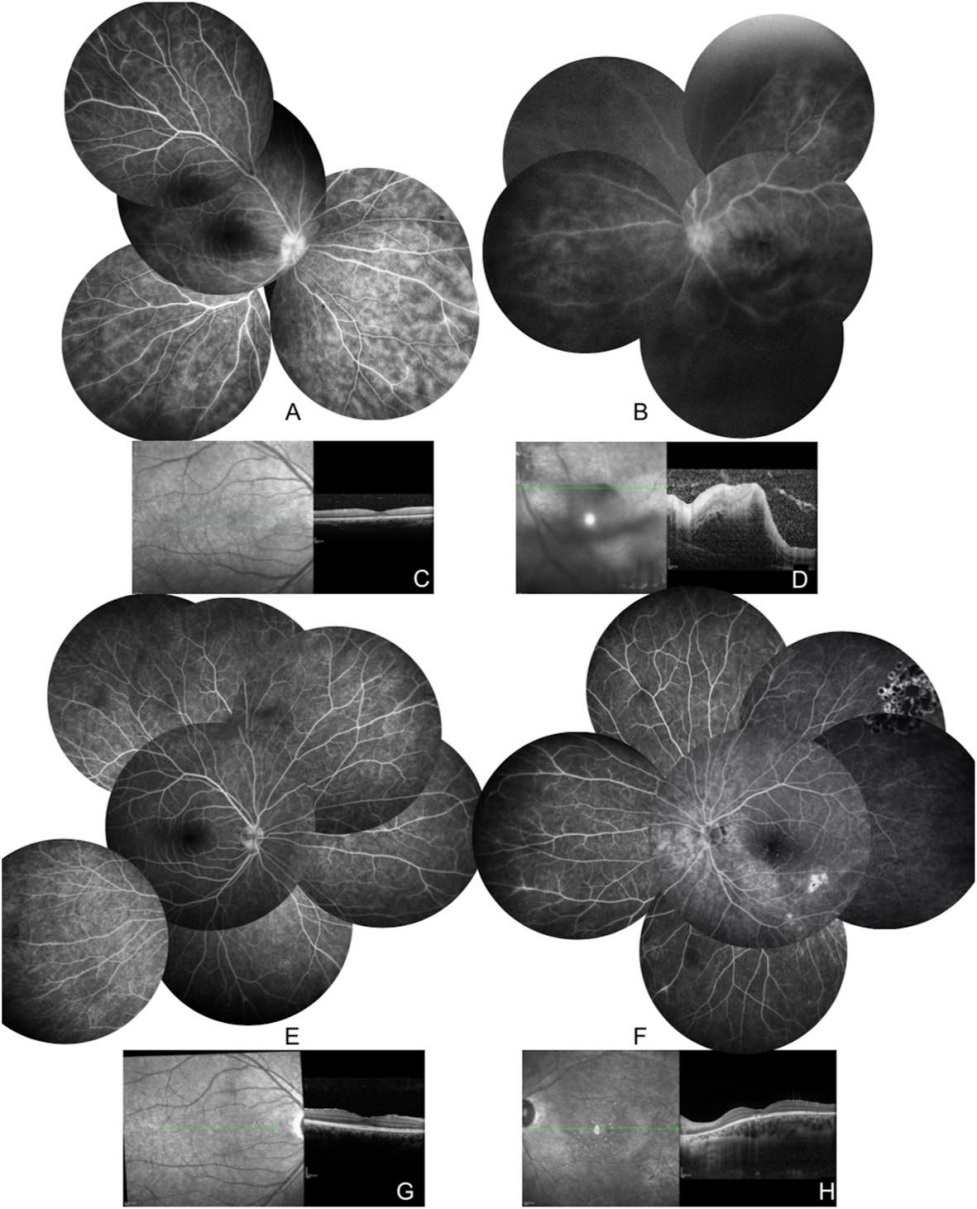
case #1. **A**-**D** Initial visit. **A**, **B** Late fluorescein angiogram showing bilateral diffuse capillaropathy and left eye vasculitis. Macular OCT shows preserved right foveal profile (**C**) while there is a massive macular edema on the left eye with retina infiltrate (**D**). **E**–**H** Last visit. Normalization of the right fluorescein angiogram (**E**), and inferotemporal pigmented epithelial atrophy in the previous zone of retinitis (**F**). Note the laser treated peripheral hole in the superotemporal periphery. Normalization of the macular OCT (**G**, **H**)

BCVA was 20/20 in the right eye and counting fingers on the left eye. Slit lamp examination and intraocular pressure were normal OU. Left fundus examination showed a dense vitreous haze associated with optic nerve and macular edema, without peripheral retinal necrosis, while OD fundus disclosed a mild vitritis. Fluorescein angiogram showed bilateral diffuse capillaropathy, papillitis and peripheral occlusive vasculitis (Fig. 1A, B). Macular OCT confirmed massive macula infiltrate and serous retinal detachment on the left eye (Fig. 1C, D). Brain magnetic resonance imaging (MRI) and lumbar puncture were normal. PBD was diagnosed and the patient was treated with intravenous pulses of methylprednisolone, followed by infliximab tapering oral prednisone associated with colchicine and azathioprine. Macular edema and serous retinal detachment completely resolved (Fig. 1E–H). Argon laser retinal photocoagulation was performed on non-perfused areas. Ocular inflammation was controlled after 3 months with this treatment regimen allowing a gradual decrease in treatment over a total of 4 years (cessation of corticosteroids and infliximab). At the last follow-up (69 months after onset) he had 20/20 OU and was still receiving colchicine and azathioprine.

## Case 2

A 9-year-old boy, from Tunisian descent, with a history of recurrent oral aphthosis, presented with a rapidly progressive bilateral visual loss with headache.

BCVA was 20/50 OD and counting fingers OS, IOP was normal OU. Biomicroscopic examination showed a massive anterior chamber reaction OU, with posterior synechiae and cyclitic membranes. A dense vitreous haze masked the details of fundus but papillary edema and retinal hemorrhages could be observed. Macular OCT was normal, while fluorescein angiography showed a venous non-occlusive vasculitis associated with diffuse peripheral capillaropathy and optic nerve leakage on both eyes.

Brain MRI was normal and lumbar puncture found an aseptic meningitis with clear aspect, normal glucose and proteins levels, but 66 cells/mm3 with predominance of neutrophils. Polymerase Chain Reaction (PCR) for herpes viruses and bacterial culture were negative. He fulfilled PEDBD criteria with oral aphthosis, ocular and neurological features.

The patient was treated with intravenous Methylprednisolone MP pulses and infliximab, followed with tapering oral prednisone associated with colchicine and azathioprine. Vitreous haze and vasculitis improved rapidly. Twelve months after onset, he recovered normal vision on the right eye, and 20/25 on his left eye, while he was still receiving prednisone, azathioprine, and colchicine. A mild papillary leakage persisted on left fluorescein angiogram.

## Case 3

**Fig. 2:**
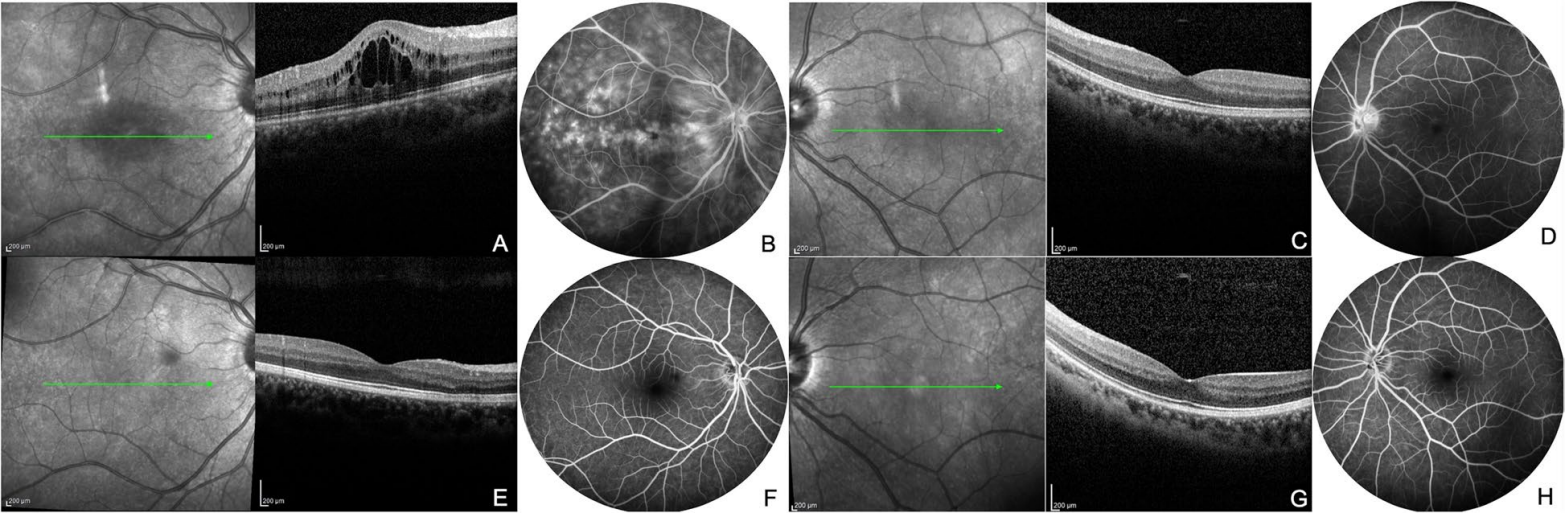
case #3. **A** Late Fluorescein angiogram showing venous non-occlusive and multifocal vasculitis. Macular edema was associated with a diffuse macular leakage on the right eye (**B**). **C** Left eye: late Fluorescein angiogram showing papillary leakage and **D** normal macular profile on OCT. **E**–**H** Twelve months after treatment initiation. Normalization of both fluorescein angiogram and macular OCT

A 14-year-old girl, from Moroccan descent had a history of encephalitis and myelitis two years before ophthalmic presentation, which was treated by intravenous MP pulses. She presented in Ophthalmology with a progressive vision loss of the right eye. BCVA was 20/32 OD and 20/20 OS. Slit lamp examination was normal and fundus showed a bilateral vitreous haze, predominating on the right eye with papillary edema. Macular OCT revealed a cystoid macular edema of the right eye (Fig. 2A), and a normal macular profile of the left eye (Fig. 2C). Fluorescein angiography revealed a venous non-occlusive and multifocal vasculitis in the right eye (Fig. 2B) bilateral papillary leakage (Fig. 2B, D) with mild peripheral vasculitis on the left eye. On the right eye, macular edema was associated with a diffuse macular leakage (Fig. 2B). Cerebrospinal fluid analysis showed an aseptic meningitis: 9 cells/mm3, glucose and protein levels were normal. PCR for herpes group virus and cultures were negative. Genetic analysis was positive for HLA B51. The patient did not fulfill PEDBD criteria as she only presented ocular and neurological features, diagnosis was retained after a multidisciplinary agreement. The patient was treated with intravenous MP pulses followed by tapering oral prednisone, associated with monthly infliximab perfusions and oral azathioprine. She recovered her full visual acuity after 3 months of treatment with a complete resolution of her macular edema and vasculitis. At last follow-up (57 months after onset), she was still in remission (Fig. 2E–H), while she was receiving infliximab, azathioprine and colchicine.

## Case 4

**Fig. 3:**
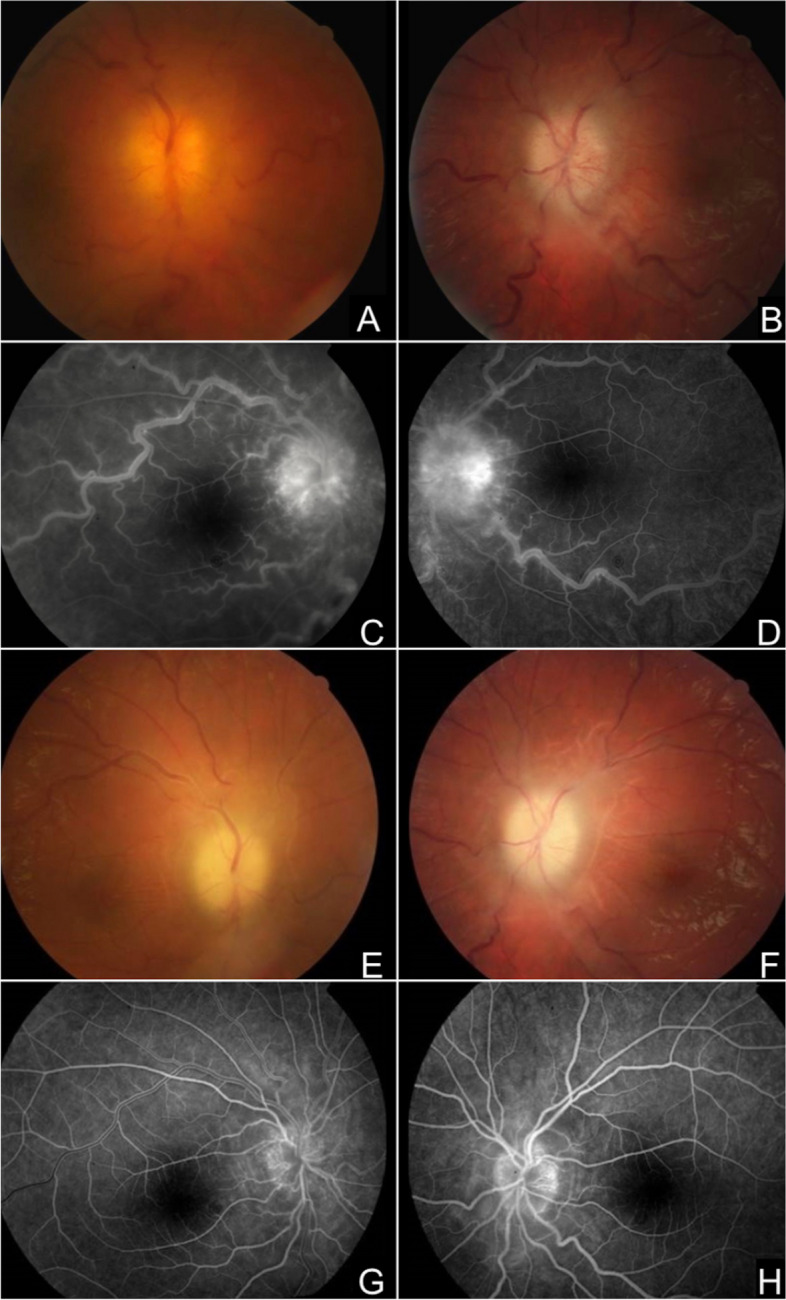
case #4. **A**, **B** Initial fundus photography showed major bilateral papilledema with venous tortuosity and bilateral vitritis. **C**, **D** Fluorescein angiography confirmed these findings. **E**–**H** Fundus photography and fluorescein angiogram performed 3 months after treatment initiation: resolution of the inflammation associated with bilateral optic atrophy

A 16-year-old boy, from Algerian descent, presented with severe general state alteration over the last 6 months, accompanied with aphthosis, headache, apathy and catatonia. He consulted at the emergency department because of red eyes and vision loss that appeared 15 days before. Ophthalmological examination showed a bilateral and severe non-granulomatous panuveitis. BCVA was limited to light perception on the right eye and 20/100 on the left eye. Initial fundus photography showed major bilateral papilledema with venous tortuosity and bilateral vitritis (Fig. 3A, B). Fluorescein angiography confirmed these findings (Fig. 3C, D).

Brain magnetic resonance imaging (MRI) showed a cerebral venous thrombophlebitis at the level of the longitudinal superior sinus and the left lateral sinus. He fulfilled PEDBD criteria with oral aphtosis, ocular, neurological and vascular features.

He was initially treated with intravenous MP pulses, infliximab perfusion, oral azathioprine and colchicine. Enoxaparine and aspirin were added for the thrombosis. One week later, intraocular inflammation and thrombophlebitis had waned, but papillary edema persisted on both eyes, with no improvement of visual acuity. After multidisciplinary discussion, he received intravenous cyclophosphamide pulses. Despite a complete resolution of the intraocular inflammation and the cerebral thrombosis, optic nerves atrophy with complete blindness (logMAR3) developed on both eyes (Fig. 3E–H).

## Discussion

In this case series of PBD, ocular manifestations were severe, with comparable phenotypes to what is usually encountered in adult BD patients [1]. All patients had posterior segment involvement, including retinal vasculitis, and required intensive systemic immunosuppressive treatments in association with systemic corticosteroids.

The prevalence of ocular manifestations in both BD and PBD is highly variable. It is reported in 9 to 76% of patients in large PBD series [5, 7, 11–15] and in 18 to 75% of adult BD series [2, 14, 16, 17].

Regarding the ocular phenotypes, the ICBD study reported that retinal vasculitis, posterior uveitis, and anterior uveitis occurred in 23%, 38%, and 40% of adult cases, respectively which seems to be comparable with what is reported in PBD [5, 7, 11, 12]. Papilledema may also be present, as in our cases. The latter can be caused by either inflammation, ischemia, or intracranial hypertension secondary to cerebral venous thrombosis [18–21]. As in adult BD, various unusual ocular presentations have been reported in PBD such as recurrent neuroretinitis [22] or immune keratitis [23]. In both adult and pediatric BD patients, bilateral panuveitis is likely to be the most frequent presentation, occurring in up to half of the patients [19, 24–26]. In both adults and children (as in our series) the macula is frequently involved [19, 24–26]. Ocular complications seem comparable in PBD and BD ocular involvement cohorts: cataract, optic atrophy and posterior synechiae are the most frequent [19, 24–26], occurring approximatively in a third to half of the patients, followed by rarer complications, such as intraocular pressure elevation, retinal detachment, neovascular glaucoma, phthisis bulbi and band keratopathy (Tables 3 and 4) [19, 24–26].

**Table 3:** Clinical characteristics of BD in children compared to BD in adult patients

	**Pediatric Behçet’s disease**							**Adult Behçet’s disease**			
	Shahram et al., 2018 [5]	Gallizzi et al., 2017 [12]	Hu et al., 2019 [13]	Nanthapisal et al., 2016 [11]	Koné-Paut et al., 2016 [7]	Gheita et al., 2019 [14]	Zou et al., 2021 [15, 17]	Gheita et al., 2019 [14]	Alpsoy et al., 2007 [16]	Davatchi et al., 2014 [9]	Zou et al., 2021^a^ [15, 17]
**Study country**	Iran	Italy	Taiwan	UK	International Multicentric	Egypt	China	Egypt	Turkey	International multicentric	China
**N patients**	204	110	55	46	156	91	69	1435	661	1278	860
**Age of onset symptoms (mean years** ± **standard deviation)**	10.5 ± 3.4	8.3 ± 4.1	11.0 (0.1–16.0)	4,9 (0.1 – 15.7)	7.4 ± 4.2	15.7 ± 2.1	NA	30.3 ± 8.1	NA	NA	36 (28 – 47)^b^
**Family history of BD (% of patient)**	10	12	NA	17	24	NA	22^c^	NA	12	11	NA
**ICBD criteria sensitivity (%)**	97	71	91	80	NA	NA	86	NA	NA	NA	86
**PEDBD criteria sensitivity (%)**	69	46	36	NA	NA	NA	56	NA	NA	NA	NA
**Diagnostic delay (mean years** ± **standard deviation)**	3.2 ± 2.6	2.9 ± 3.6	NA	3.7 (0.3—13.5)	6.0 ± 3.5	NA	NA	NA	5.3 ± 6.3	NA	NA
**M/F rate (%)**	50/50	56/44	41/59	48/52	50/50	69/31	55/45	72/28	53/47	57/43	54/46
**Ocular involvement (%)**	66	44	27	9	45	76	9	75	29	55	18
**Anterior uveitis (%)**	52	26	NA	4	30	NA	1	NA	NA	40	2
**Posterior uveitis (%)**	58	13	NA	0	28	NA	NA	NA	NA	38	NA
**Retinal vasculitis (%)**	40	9	NA	0	17	NA	NA	NA	NA	23	NA

*ICBD* International Criteria for Behçet’s Disease [9], *PEDBD* PEDiatric Behçet’s Disease [7], *BD* Behçet’s disease, *M* Male, *F* Female, *UK* United kingdom, *NA* Not available

^a^This cohort includes 69 cases of juvenile onset Behçet’s disease

^b^Median age (Interquartile range)

^c^Familiy history of oral aphtosis

**Table 4:** Frequencies of ocular features, complications and systemic associations in uveitis associated with BD in children compared to BD in adult

	**Pediatric Behçet’s disease**		**Adult Behçet’s disease**	
Clinical features (% of affected eyes)	Tugal-Tutkun et al., 2003, Turkey *N* = 36 [25]	Citirik et al., 2009, Turkey *N* = 34 [24]	Tugal-Tutkun et al., 2004, Turkey *N* = 880 [19]	Yang et al., 2008, China *N* = 485 [26]
Bilateral uveitis	83	44	78	77
Panuveitis	86	53	60	69
Anterior uveitis	14	15	11	7
Retinal hemorrhage	NA	NA	27	NA
Retinal vasculitis	86	NA	89	81
Retinal vein occlusion	3	6	NA	22
Retinitis	75	NA	52	53
Papillitis	NA	NA	6	NA
Disk neovascularization	3	3	NA	NA
Maculopathy	58	NA	NA	NA
Macular edema	NA	15	45	34
Hypopion	25	NA	12	32
Elevated IOP	11	21	14	31
Cataract	50	59	39	77
Posterior synechiae	31	24	26	NA
Optic atrophy	47	29	24	NA
Macular degeneration	NA	NA	19	NA
Epiretinal membrane	NA	NA	17	10
Retinal tear	6	NA	1	2
Retinal detachment	6	NA	1	16
Exsudative retinal detachment	11	NA	NA	10
Pars plana exsudates	17	NA	NA	NA
Neovascular glaucoma	3	NA	1	NA
Phtisis	3	3	2	NA
Band keratopathy	3	NA	NA	NA
Oral aphtosis	100	NA	100	100
Genital ulcer	61	NA	60	58
Arthritis	22	NA	34	39
Skin involvement	72	NA	55	78
Neurological features	8	NA	4	1
Vascular features	3	NA	5	16
Positive pathergy test	54	NA	NA	NA

*NA* Not available, *BD* Behçet’s disease, *IOP* Intraocular Pressure

### Epidemiology of pediatric BD

Age at onset of symptoms also varies among the different PBD cohorts. Koné-Paut et al*.* and Nanthapisal et al*.*reported a mean age at onset of BD of 4.9-year-old (0.1 to 15.7 y.o.) and 7.4 ± 4.2 y.o., respectively [2, 7]. In these two studies, family history was presentin 17% and 24% of patients, respectively, a higher rate than in adult BD cohorts, suggesting a stronger familial aggregation in children [2, 7, 11, 14]. Female/male ratio seems similar in BD and PBD [2, 5, 7, 9, 11–17, 13, 14, 16, 27]. Geographical location and ethnic origins may be involved in clinical phenotypes seen in PBD cohorts [2]. For instance, the prevalence of ocular involvement reported in Iran and Egypt(66% and 76% respectively) [5, 14], seems higher than what was reported in Italy, Taiwan, China and UK (44%, 27%, 9% and 9% respectively) [11–13, 15]. BD is typically considered as a polygenic disorder. Zhou et al. reported 6 families with early onset auto-inflammatory disease caused by heterozygous mutation in the TNFAIP3 gene coding for A20 proteins with clinical manifestations mimicking BD. However, a mutation in A20 protein was identified in only one patient in a large BD genome-wide association study [28].

### Diagnostic delay and criteria

As seen in our study, delay of diagnosis may be an important issue in PBD, especially when ocular manifestations reveal the disease, as children may not complain in cases of unilateral and painless ocular involvement. As in other inflammatory diseases, diagnostic delay may impact the severity of clinical presentation. In published literature, diagnostic delays for PBD vary from 2.9 ± 3.6 years [12], to 6.0 ± 3.5 years [7]. However, this long diagnostic delay is also found in adults [16].

In our study, three patients met the PEDBD criteria, while one patient was diagnosed on the combination of typical neurological features (cerebellar peduncle) and retinal vasculitis, both of which are highly suggestive of Behçet's disease [29]. However, three patients met the ICBD criteria designed for adult BD [2]. As in our series, in previously published PBD cohorts, ICBD criteria are constantly more sensitive than PEDBD criteria (71 to 97% versus 36 to 69%, respectively) [5, 12, 13, 15]. ICBD and PEDBD criteria comprise 7 and 6 items, respectively, that are weighted in ICBD classification while they’re not in PEDBD criteria [2, 7]. Thus, the presence of uveitis and oral aphtosis is sufficient to diagnose PBD with ICBD but not with PEDBD criteria. ICBD criteria have been developed from larger cohorts comprising both adult and children originating from various countries, with the underlying hypothesis that adult and pediatric disease are similar [2]. While in most of the cases, oral or genital ulcerations are the first symptoms of the disease, scarce information is available about the referring symptom in neither PBD studies nor BD studies [2, 5, 7, 11–17, 19, 24–26].

In cases of isolated ophthalmic or atypical presentations, other cause of posterior uveitis may be considered in the pediatric context, including infectious diseases, such as toxoplasmosis, toxocarosis, and herpesviridae infections, that can be associated with vitritis and retinal necrosis [30]; inflammatory diseases such as (but not limited to) sarcoidosis or Blau Syndrome that can cause posterior uveitis with retinal vasculitis [31]. Uveitis associated with tubulointerstitial nephritis and uveitis syndrome and juvenile idiopathic arthritis are usually limited to the anterior segment [32, 33].

### Treatments

Contrarily to adult BD, there is no recommendation available to treat ocular manifestation in PBD. As in our series, other teams used European alliance of associations for rheumatology (EULAR) recommendation derived treatment protocols [11–14, 34]. Intravenous high dose corticosteroids are helpful in the acute phase, but long-term oral steroids are used with caution in children essentially because of their impact on children’s growth [35]. Immunosuppressive therapies are recommended in combination with steroids in posterior uveitis [34], but data on their efficacy in PBD are scarce. Some small case series reported the efficacy of treatments such as methotrexate, cyclosporine, chlorambucil, azathioprine, sulfasalazine, cyclophosphamide or thalidomide in this context [11–13, 25, 36]. Regarding biotherapies, anti-TNF α, mainly infliximab, which is recommended in EULAR guidelines for severe cases has been reported in PBD series [11, 12, 30, 37]. Serious side effects with long-term anti-TNF α use in non-infectious pediatric uveitis remains rare, infliximab antibodies is the most frequent issue [30, 38].

Interferon have been reported in PBD case reports or small case series in association with conventional immunosuppressive treatments for severe and relapsing forms, but adverse effects are frequent (flu-like syndrome especially) and may be severe (lymphopenia, neutropenia, depression) [36, 30, 39, 40]. In our experience, in addition to the choice of the drug itself, the most important elements in managing PBD and other severe inflammatory ocular conditions in children are i) a close collaboration between pediatricians and ophthalmologists to manage systemic manifestations, optimize systemic treatment while monitoring side effects and adherence, and ii) to provide educational support to patients and their family, in order for them to be involved in the therapeutic project [41].

### Prognosis

As in adults, visual prognosis of ocular involvement in PBD is guarded. In a cohort of 36 PBD patients with 66 eyes affected by uveitis, and a mean follow up of 84.7 ± 91.5, Tugal-Tuktun et al. reported 23% of eyes with a severe and irreversible visual loss (BCVA below 20/200), and 17% of patients with legal blindness (BCVA below 20/200 on the best eye) [25]. Koné-Paut et al. reported a BCVA < 20/200 in 19% of PBD patients with uveitis in at least one eye, and 3% of patients with legal blindness [42]. In recent adult BD cohorts with uveitis, the proportion of patient with at least one eye with BCVA < 20/200 varies between 28 and 30% [43, 44]. In PBD, the most frequent cause of legal blindness.

## Conclusions

To summarize, as in adult, ocular involvement is a frequent and potentially blinding manifestation of BD in children. According to our case series and the literature, pediatric ocular phenotypes seem comparable to those observed in adults. On the other hand, it seems important not to omit the search for a family history that may raise suspicion of an autoinflammatory disease in this specific context. A multidisciplinary evaluation with an early ophthalmological evaluation is absolutely required for any suspicion of PBD.

## Data Availability

The datasets supporting the conclusions of this article are included in the tables and the figures.

## Abbreviations

PBD: Pediatric Behçet disease
BD: Behçet disease
PEDBD: Pediatric Behçet’s Disease Study
ICBD: International Criteria for Behçet’s Disease Study
BCVA: Best-corrected visual acuity
SUN: Standardization of Uveitis Nomenclature
OCT: Optical Coherence Tomography
IRB: Institutional Review Board
MEFV: Familial Mediterranean Fever
PFAPA: Periodic fever, aphthous stomatitis, pharyngitis, adenitis
MRI: Magnetic resonance imaging
PCR: Polymerase Chain Reaction
MP: Methylprednisolone
EULAR: European alliance of associations for rheumatology
